# ORF8 ∆382 Mutation: A Possible Viral Prognostic Biomarker for the Severity of the COVID-19

**DOI:** 10.52547/ibj.25.4.308

**Published:** 2021-05-17

**Authors:** Alireza Tabibzadeh, Maryam Esghaei, Farhad Zamani, Saber Soltani, Parastoo Yousefi, Mohammad Hadi Karbalaie Niya

**Affiliations:** 1 Department of Virology, Faculty of Medicine, Iran University of Medical Sciences, Tehran, Iran;; 2 Gastrointestinal and Liver Diseases Research Center, Iran University of Medical Sciences, Tehran, Iran;; 3 Department of Virology, School of Public Health, Tehran University of Medical Sciences, Tehran, Iran

**To the editor:**

Since the initial onset of the current severe acute respiratory syndrome coronavirus 2 (SARS-CoV-2) pandemic from Wuhan, China in December 2019, the virus is now spread all around the world, and the disease is named COVID-19. Based on the WHO report on April 18, 2021, there are now more than 140 million confirmed cases and three millions deaths due to this disease^[^^1^^]^. The virus pathogenesis, clinical features, and therapeutic approaches have comprehensively been reviewed^[^^2^^]^. In addition, it has been indicated that patients' medical background and the age factor are associated with the mortality rate^[^^2^^]^. Regardless of the clinical presentations and the disease mortality, the mental effects of the current pandemic among medical staff and the general population are critically important, and the pandemic might trigger stress, anxiety, and depression^[^^3^^]^.

In a research conducted by Derry *et al.*^[^^4^^]^, it has been suggested that in cancerous patients, understanding of the disease prognosis could be helpful for therapeutic decision making and mental health. Despite a wide range of differences between cancer and COVID-19 illnesses, introducing a prognostic marker for COVID-19 could be helpful. Furthermore, this marker could be applied to predict possible admission to ICU and/or in case of a severe or mild disease. Excessive number of patients who required ICU admission have been reported in countries with COVID-19 crises^[^^5^^]^. 

SARS-CoV-2 genome encodes non-structural proteins (ORF1a/1b) and several structural proteins, including spike protein (S), envelope protein ([E), membrane protein (M), nucleocapsid protein (N), and accessory proteins (3a, 6, 7a, 7b, 8, and 10)^[^^2^^]^. As mutations could accelerate the virus adoption and evolution, variant detection is popular in this context^[^^6^^]^. In this regards, SARS-CoV investigations in 2002-2003 illuminated that ORF8 mutations might be a reason for the virus attenuation^[^^7^^]^. The exact role of the ORF 8 is unclear but seems to be important in virus infection cycle and virus evasion from immune system^[^^8^^]^. This mutation, the SARS-CoV-2 ORF8 deletion, was initially reported in Singapore and then in travelers from Wuhan to Taiwan^[^^6^^,^^9^^]^. These mutations in ORF 8 were also reported in Bangladesh, Australia, and Spain^[^^6^^,^^8^^,^^9^^]^. Recently, in one study by Young and colleges^[^^8^^]^, the clinical features of the patients with and without ORF8 ∆382 mutations have been reported. ORF8 ∆382 mutant detected by hemi-nested PCR illustrates the lower rate for hypoxia development and requirement for supplemental oxygen in comparison with wild type or co-infected patients.

Considering the importance of introducing a prognostic marker for COVID-19 and the ORF8 ∆382 assessment, as Young *et al.*^[^^8^^]^ illustrated, it could have a potential to be evaluated as a prognostic marker for COVID-19 severity and the hypoxia development in SARS-CoV-2-infected patients. In the assessment of 131 patients, 22% had only ORF8 ∆382 strain and 8% were infected by both wild type and ORF8 ∆382. Using such a prognostic marker could provide a more clear picture of the patient’s clinical course of the disease and outcome. Moreover, this potential prognostic marker could be useful for making decision on therapeutic options and ICU admission. It is noteworthy that the Young *et al.*’s^[^^8^^]^ study provides limited data in this field, and introducing an ORF8 ∆382 mutation as a prognostic marker critically needs further comprehensive investigations. 

## CONFLICT OF INTEREST.

None declared.
